# Cannabidiol effects in stem cells: A systematic review

**DOI:** 10.1002/biof.2148

**Published:** 2024-12-09

**Authors:** Cristina Mesas, Javier Moreno, Kevin Doello, Mercedes Peña, Juan M. López‐Romero, Jose Prados, Consolación Melguizo

**Affiliations:** ^1^ Institute of Biopathology and Regenerative Medicine (IBIMER), Center of Biomedical Research (CIBM) University of Granada Granada Spain; ^2^ Instituto de Investigación Biosanitaria de Granada, (ibs.GRANADA) Granada Spain; ^3^ Service of Medical Oncology Hospital Virgen de las Nieves Granada Spain; ^4^ Department of Organic Chemistry, Faculty of Sciences University of Malaga Málaga Spain; ^5^ Department of Anatomy and Embryology, Faculty of Medicine University of Granada Granada Spain

**Keywords:** stem cellscancer stem cells, CBD, in vitro, in vivo

## Abstract

Stem cells play a critical role in human tissue regeneration and repair. In addition, cancer stem cells (CSCs), subpopulations of cancer cells sharing similar characteristics as normal stem cells, are responsible for tumor metastasis and resistance to chemo‐ and radiotherapy and to tumor relapse. Interestingly, all stem cells have cannabinoid receptors, such as cannabidiol (CBD), that perform biological functions. The aim of this systematic review was to analyze the effect of CBD on both somatic stem cells (SSCs) and CSCs. Of the 276 articles analyzed, 38 were selected according to the inclusion and exclusion criteria. A total of 27 studied the effect of CBD on SSCs, finding that 44% focused on CBD differentiation effect and 56% on its protective activity. On the other hand, 11 articles looked at the effect of CBD on CSCs, including glioblastoma (64%), lung cancer (27%), and breast cancer (only one article). Our results showed that CBD exerted a differentiating and protective effect on SCCs. In addition, this molecule demonstrated an antiproliferative effect on some CSCs, although most of the analyses were performed in vitro. Therefore, although in vivo studies should be necessary to justify its clinical use, CBD and its receptors could be a specific target to act on both SSCs and CSCs.

## Graphical abstract

CBD acts as a differentiator and protector of CSCs and exerts a significant antitumor effect against CSCs indicating a great potential as a new therapeutic strategy in regenerative medicine and oncology.

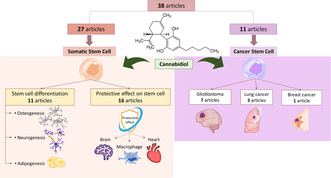

**Abbreviations**
AD: adipogenesis
AD‐MSC: adipose‐derived mesenchymal stem cells
alloHCT: allogenic hematopoietic cell transplantation)
ALS: amyothrophic lateral sclerosis
AMSC: adipose mesenchymal stem cell
APD: AP duration
APL: alkaline phosphatase
APSC: apical papilla stem cell
BM‐MSC: bone marrow mesenchymal stem cells
BRDU: bromodeoxyuridine
CBD: cannabidiol
CM: commercial
CSCs: cancer stem cells
DFC: 2′,7'dichlorofluoresceindiacetate
DFSC: dental follicle stem cell
DNPH: 2,4‐dinitrophenylhydrazine
DPSC: dental pulp stem cell
GBM: glioblastoma multiforme
GICs: glioma initiating cells
GSC: glioblastoma stem cells
GVHD: graft‐versus‐host disease
hGMSC: human gingivial mesenchymal stem cell
hiPSC‐CM: human induced pluripotent stem cell‐derived cardiomyocytes
HNP: hybrid nanoparticles
HO‐1: heme oxigenase‐1
HPLDSC: human peridontal ligament stem cells
IC_50_
: half‐maximal inhibitory concentration
i.p: intraperitoneal
KO: knock‐out
LPS: lipopolysaccharides
MOR: moringin
MPLA: monophosphoryl lipid A
NP: neuroprotective
OSP: oxidative stress protection
OTG: osteogenesis
OTP: osteoprotection
OTR: osteoregeneratio)
PLGA: poly(lactic‐co‐glycolic acid)
PE: piperazine erastine
PN: proneural
PPAR‐γ: peroxisome proliferator‐activated receptor gamma
rCNC: rat cortical neural cells
RS: reticulum stress
RXR‐α: retinoid X receptor alpha
SSCs: somatic stem cells
SSPC: skeletal stem and progenitor cell
TBARS: triobarbituric acid‐reactive substances
TGF‐β: transforming growth factor beta
THC: tetrahydrocannabinol
TMZ: temozolomide

## 1 INTRODUCTION

Cannabidiol (CBD) is an organic molecule that belongs to the cannabinoid family, a group of terpenophenols that can be isolated from *Cannabis sativa L*. and are characterized by their potential in the treatment of certain diseases, especially neuropathies by an interaction with CB1 and CB2 receptors.^1^, ^2^, ^3^ CBD has a huge advantage over other cannabinoids, such as tetrahydrocannabinol (THC), in that it does not have a high affinity for CB1 and CB2 receptors expressed on neural somatic cells, which disables the development of psychotropic effect in the patient.^4^ Even so, there are currently numerous studies that demonstrate the capacity of CBD as a drug against numerous pathologies, where its anti‐inflammatory and antioxidative effect stands out.^5^ In addition, it has been shown to exert an antitumor effect, either by exerting cytotoxic, antiproliferative, or antimigratory activity.^6^, ^7^ Interestingly, CBD receptors have been described on the somatic stem cells (SSCs) and even in special stem cells present in tumor masses (CSCs). Some of the receptors that are expressed in human bone marrow mesenchymal stem cells (hBM‐MSCs)^8^ and adipose tissue mesenchymal stem cell (AMSC) are the CBD receptors CB1 and CB2.^9^ CSC such glioblastoma stem cell can also produce this receptors.^10^ Additionally, GPR55 and TRPV1 receptors can be activated by CBD, expressed both in SSCs^11^ and CSC like glioblastoma stem cells.^10^, ^12^ Lastly, nuclear receptor PPAR‐γ is also expressed in stem cells.^13^, ^14^ CBD has an antitumoral effect due to the activation of these receptors, reducing metastatic capacity and proliferation or activating apoptosis.^7^ On the other hand, CBD receptors also control the differentiation, proliferation and self‐renewal in SSCs.^15^

Nowadays, there is only a few studies that directly investigate the effect of CBD on CSC, but its potential resides on its ability to interact and modify signaling pathways involved in the stemness of CSC. For example, CBD reduces the Wnt/ β‐catenin pathway, which is closely related to the drug resistance of most of CSC, including ovarian cancer stem cells (OCSC).^16^ Cellular drug resistance can also be mitigated through increasing TRPV2 protein activity.^17^ Additionally, it reduces CD44 levels and NF‐κB signal pathway implied on CSC development.^16^ CBD could also reduce the abnormal Id1 levels of CSC by modifying p38 signaling, triggering a regulation in other pathways involved in tumor development such STAT3 and TGF‐β.^18^, ^19^

Stem cells (SCs), a collection of undifferentiated cells that exhibit self‐renewal ability, clonality and potentiality to differentiate into other cell types,^20^, ^21^ have been classically classified in totipotent, pluripotent, multipotent, oligopotent, and unipotent cells. Most stem cells are found in the embryo, but stem cells from the adult organism are an interesting target for tissue repair and renewal.^20^, ^22^, ^23^ In addition, CSCs, a group of tumor‐mass cells that ensure self‐renewal, have been described in most cancers.^24^, ^25^, ^26^ These cells are characterized by drug resistance mediated by some mechanism such as increased DNA repair capacity, reduced apoptotic capacity, phenotypic plasticity, or increased production of drug transport channels that increase their outflow.^24^

In this context, the presence of SSCs in adult tissue and their ability to proliferate and differentiate gives them enormous clinical potential. For example, MSC can be grafted into a wound to stimulate others stem cells present in the body and increase their migration and differentiation in this area.^27^, ^28^ Some cardiac diseases have been treated using stem cells to develop cardiomyocytes with optimal electrochemical characteristics but without remarkable results.^29^, ^30^ In addition, hematopoietic stem cell transplantation (HSC) has been applied in leukemia or anemia^31^ and stem cell therapy has been used in neurological diseases such as Parkinson's, Huntington's, dementia, sclerosis, and among others.^32^, ^33^ Other applications of stem cells, such as diabetes mellitus type 1 treatment, infertility by differentiating stem cells into gametes, and spinal cord injury treatment, are being studied.^31^, ^34^, ^35^ On the other hand, the CSCs present in a tumor mass constitute another new opportunity for cancer treatment. In fact, these cells are responsible for the high relapse rates of many cancers such as glioblastoma, lung cancer, pancreatic cancer, and among others, despite surgical, radiotherapeutic, and chemotherapeutic treatment. Thus, the use of molecules focused on modulating the CSC behavior represents a great opportunity for cancer therapy. Experimental therapies such as Rovalpituzumab‐tesirine, which is capable of destroying CSCs with delta ligand, or Disulfiram, which inhibits (aldehyde dehydrogenase) ALDH, are the basis of recent clinical trials^36^ and drugs such as Vismodegib, which blocks the Sonic Hedgehog pathway in CSCs, is a potent new therapeutic weapon against metastatic basal cell carcinoma.^37^ The demonstrated presence of CBD receptors in both CCS and CSC opens up the possibility that this type of cell can be modulated by CBD toward both a differentiation process and a proliferation control process, which could be useful from a therapeutic point of view. In these cases, CBD does not interact directly with CB1 and CB2 receptors, but inducing an allosteric modulation of the receptor and their affinity with others compounds like endocannabinoids.^38^

Thus, the objective of this systematic review was to discuss about the effect of CBD over SSC and against CSC and make comparisons in experimental designs and results obtained.

## 2 METHODS

First, this systematic review has been registered in the OSF database, on February 7, 2024 (https://doi.org/10.17605/OSF.IO/NB3G).

### 2.1 Study eligibility and data sources

The objective of this systematic review has been to analyze all the articles published to date that study the effect of CBD on stem cells, both CSC and SSC.

To do this, first a bibliographical search was carried out in four different electronic databases: PubMed, SCOPUS, Web of Science and Cochrane. To perform the search in PubMed, the following “MeSH” terms were used: “cannabidiol” and “stem cells”, with the formula obtained: (“cannabidiol” [MeSH Terms] OR “cannabidiol” [All Fields] OR “cannabidiolic” [All Fields]) AND (“stem cells” [MeSH Terms] OR (“stem” [All Fields] AND “cells” [All Fields]) OR “stem cells” [All Fields] OR (“stem” [All Fields] AND “cell” [All Fields]) OR “stem cell” [All Fields]). In the case of the other databases, this formula was adapted. Finally, an additional literature search was conducted to include additional articles of interest. Moreover, this systematic review has followed PRISMA guide to guarantee its correct execution.^39^

### 2.2 Inclusion criteria

Because this systematic review aims to analyze all articles that study the effect of CBD on stem cells, no restriction has been made by publication date. All articles that have carried out in vitro, in vivo or clinical trials have been included, as well as studies in vitro or in vivo carried out from patient biopsies or from established cell lines. Both studies on CSCs and CCS have been included. Articles that obtained CBD from plant extracts or from a commercial house have been included.

After reviewing the bibliography of the articles included in the systematic review, those that met the inclusion and exclusion criteria were also added to this systematic review.

### 2.3 Exclusion criteria

The main exclusion criterion has been that no studies were specifically carried out on stem cells. Articles that studied the antitumor activity of CBD but did not carry out specific studies on CSCs were also excluded. Those articles that tested other types of cannabinoids other than CBD were excluded. However, those that compared different types of cannabinoids including CBD were included. Regarding language, articles that were written in a language other than Spanish, English, or French were also excluded.

### 2.4 Study selection

**FIGURE 1:**
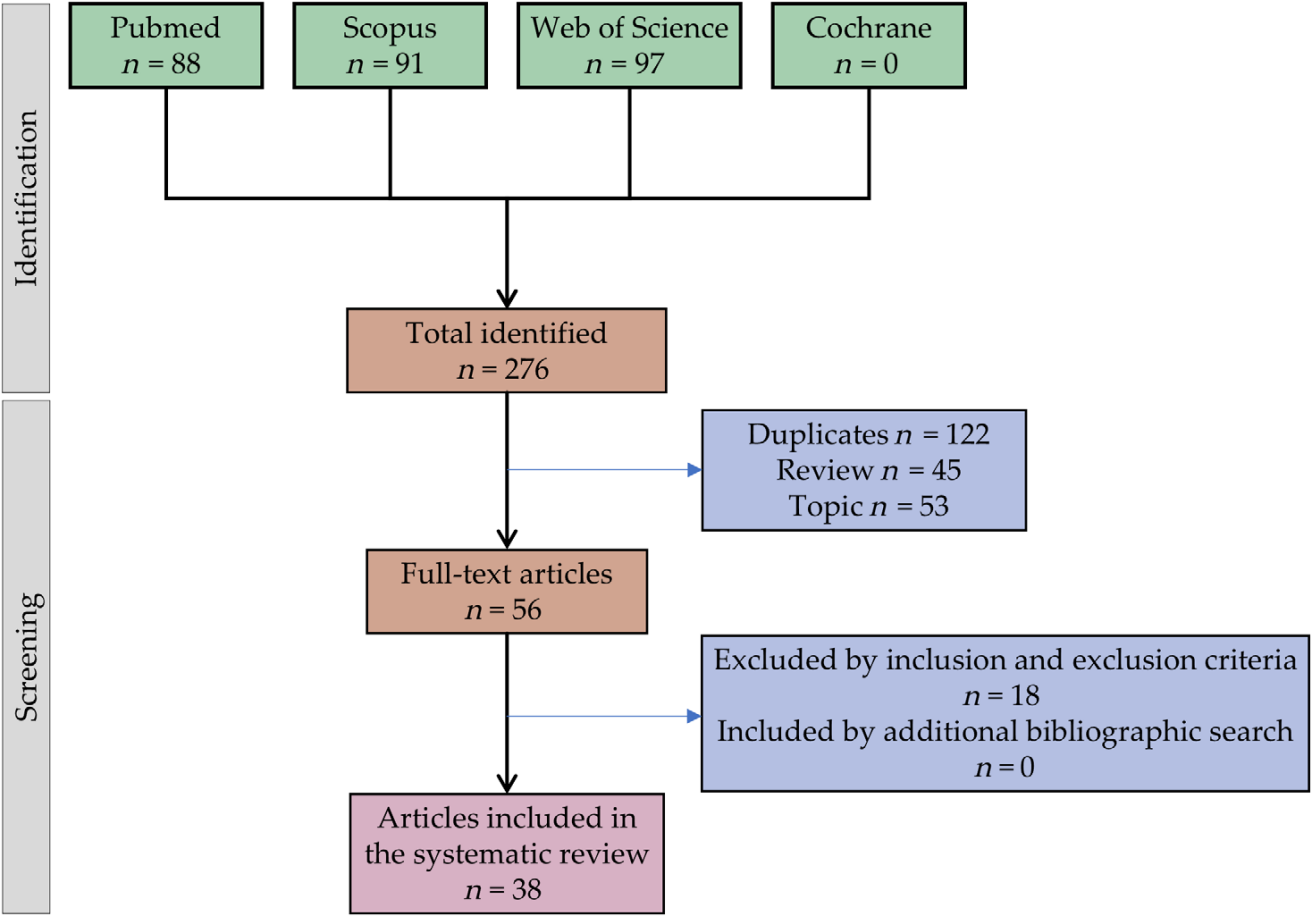
Flow diagram illustrating the search and selection process for articles included in the systematic review.

Authors CM and JM carried out the first bibliographic search independently and agreed on the search formula for each database obtained 276 articles. Once the articles were obtained, those that were not original articles were not freely accessible, were repeated, and were excluded, obtaining a total of 56 articles. In the second step of the procedure, independently, CM and JM carried out a detailed reading of the articles, excluding those that did not meet the inclusion and exclusion criteria, obtaining a total of 38 articles, these being the ones that were finally analyzed in this systematic review (Figure 1). It is noteworthy that no additional articles were included after performing the additional literature search.

### 2.5 Data extraction

Following the procedure described, C.M. and J.M carried out the procedure independently. According to Cohen's Kappa statistic test,^40^ there was good correlation between C.M. and J.M. Any disagreements were resolved through discussion until a consensus was reached. Otherwise, a third experienced author made the final decision (M.P). Finally, each article was subjected to a quality test independently by C.M. and J.M. This quality test has two parts, the first consists of general filters on cell lines and patient biopsies (score ≥5). Articles that did not reach this score were excluded. The second phase consisted of questions on the isolation and study of CSCs and the effect of CBD on them. The articles were classified according to the score obtained: low quality (score 0–5), medium quality (score 6–15), and high quality (score 16–20). No article was excluded by this quality criterion, with all included articles obtaining high quality ratings. Therefore, the 38 articles have been summarized in tables specifying the methodology and main results obtained, both in SSC (Tables 1 and 2) and on CSC (Table 3).

**TABLE 1:** Effect of CBD on SSC differentiation.

Aim	Ref	Stem cell isolation	Methodology	Results
OTG	Kamali et al.^41^	MSCs obtained from the scaffold samples after the in vivo experiment	In vitro: Study of porosity, biodegradation and RT‐qPCR. In vivo: Adult male Wistar rats with undergone forearm ostectomy. CBD‐free‐G/nHAP or CBD‐PLGA‐G/nHAp scaffolds with 500 μL of a CBD (1 mg/mL) were added, except for controls.	↑ motility and migration of mesenchymal stem cells in vitro. ↑ osteoregenerative capacity of the scaffold in an in vivo model.
	Li et al.^42^	BM‐MSC obtained from the femur of male C57BL/J6 mice.	In vitro: Cytotoxicity study with CCK‐8. Measurement of alkaline phosphatase activity and analysis by RT‐qPCR and Western Blot of genes and proteins involved in osteogenesis and inflammation.	No cytotoxic effect ↓ expression of TNF‐α and IL‐6 (2.5–5 μM CBD). ↓ inflammatory response and enhancing osteogenesis via the CB2/p38 MAPK signaling pathway. ↑ Runx2, osteocalcin and CB2.
	Petrescu et al.^43^	APSC, DFSC and DPSC isolated from obtained from samples of human apical papilla, dental follicule and dental pulp respectively.	In vitro: Cell viability assay with Alamar blue, in addition to a mineralization assay with alizarin red assay and RT‐qPCR of some osteogenesis‐related genes.	↑ cell proliferation in the DPSC and APSC cell lines. ↑ expression of osteogenic proteins in APSCs and mineralization in the DFSC cell line. ↑ the transcription of osteogenic genes such as osteonectin, osteopontin and osteocalcin.
	Schmuhl et al.^44^	AD‐MSC obtained from human subcutaneous adipose tissue biopsies.	In vitro: Cell migration assay and a wound healing assay were performed. For osteogenesis, RT‐qPCR and Western blotting were performed, an ALP activity assay and a mineralization assay by means of an o‐cresolphtalein complexome assay.	↑ migration via p42/44 MAPK pathway ↑ ALP activity and mineralization. ↑ BMP‐2, BMP7, colony stimulating factor 2, msh homebox‐1, enamelin, VEGFB.
	Yu et al.^45^	DPSC obtained from human wisdom teeth and premolars.	In vitro: Cell proliferation assay was performed using CCK‐8. On the other hand, cell migration was studied by wound healing assay. Finally, the expression and activity of some osteogenic genes was studied by ALP activity assay, RT‐qPCR amplification and Western blot analysis.	↑ cell proliferation at CBD concentrations between 0.1–12.5 μM. ↑ Matrix mineralization after day 14. ↑ migratory capacity of DPSCs at 2.5 μM CBD dose. ↑ CB1 and CB2 receptors in DPSC. ↓ negative effect of TNF‐a on the migration, viability and differentiation of DPSCs by ↓ IL6 and IL1β at concentrations of 2.5 μM.
	Ihejirika‐Lomedico et al.^46^	In vitro SSPC cells obtained from human cancellous bone.	In vitro: Cell viability assay by PrestoBlue kit, proliferation by BrdU assay and osteogenic gene expression analysis by RT‐qPCR. In vivo: Male C57BL6 with a fracture in the anterolateral area of the femur. Treatments were divided into CTL, CBD pre‐fracture, CBD post‐fracture and CBD with osteoporosis induced by fluoxetine or by ovarectomy. CBD administration at a daily rate of 5 mg/kg of animal by implanted osmotic pump.	In vitro: ↑ cell viability at 1 μM CBD and 2 μM ↑ expression of Runx2 and Osterix. In vivo: No differences were observed in bone stiffness and bone fracture repair. CBD is able to prevent negative effects in a fluoxetine and ovariectomy induced osteoporosis ↓ the delayed effect on bone regeneration in mice with this disease.
NG	Fihurka et al.^47^	BM‐MSC obtained from YAC128 mice	In vitro: Evaluation of cell viability with trypan blue and anti‐inflammatory study by evaluation of IL‐6 expression by ELISA after cellular exposure to LPS. Gene expression study by RT‐qPCR of the *htt* gene.	↓ inflammation by ↓ IL‐6 production. ↑ cell viability. ↓ *htt* gene in CBD treatment. ↓↓ *htt* gene in HNP‐co‐(CBD + siRNA) treatment.
	Razavi et al.^48^		In vivo: Adult male albino Wistar rats. CBD dissolved in 10% DMSO and 90% phosphate buffer, injecting 5 μL of the solution into the lateral ventricle of the brain (50 μg CBD / 5 μL). After the experiment, the sample was excised and microinjected, and a fluorescence immunohistochemistry assay was performed.	↑ cell proliferation in abstinence rats. ↑ neuroblast by a ↑ neurogenesis in abstinence rats (↑DCX‐positive cells). = proliferation and neurogenesis between CBD in abstinence rats and DMSO in normal rats. ↓ neuronal degeneration and ↓ number of dark neurons by CBD treatment in abstinence rats. = number of dark neurons between CBD in abstinence rats and DMSO in normal rats.
	Lanza Cariccio et al.^49^	Human hPDLSC cells from periodontal ligament tissues.	In vitro: Phenotypic evaluation by stem cell flow cytometry and microscopy. In addition, cytotoxicity was evaluated by MTT assay and gene expression by RT‐qPCR. In addition, an immunofluorescence assay has been performed.	No toxic effect of CBD + MOR solution. Cytoskeletal remodeling on the cells. ↑ GAP43 and Nestin in CBD + MOR treatment. ↑ BDNF and GFAP neuronal and glial markers. ↑ neuronal survival and differentiation capacity by PI3K/Akt/mTOR activation. Enhanced effect with MOR and CBD co‐treatment.
AD	Fellous et al.^50^	BM‐MSC cells obtained from the femur of female C57BL/6J mice.	In vitro: Fibroblast formation capacity and MTT cytotoxicity assay. Study of the amount of adiponectin by ELISA. Glucose elicitation capacity assay. Western blot.	↑ fibroblast colony formation capacity of BM‐MSC at 5 μM CBD by a CB2‐activation pathway. ↑ viability of BM‐MSC cells and induction of adipogenesis by increasing the adiponectin levels in combination with CBG due to a partially‐dependent PPARγ activation at 5 μM CBD. ↑ *Fabp4* gene expression (5 μM CBD). *Glut* and *Scd* levels ↑ 5 μM CBD and ↑↑ 5 μM CBD + 5 μM CBG.
	Chang et al.^51^	hAD‐MSC (hTERT), hBM‐MSC and mBM‐MSC purchased.	In vitro: Real‐time RT‐qPCR and assessment of PPAR‐Y and RXRa activity using a luciferase assay.	↑PPAR‐γ activation at CBD concentrations above 100 nM, but no effect on RXRa. ↑ lipid accumulation in hMSC (dose‐dependent CBD, above 2.5 μM) and mMSC (above 5 μM). ↑ adipogenic genes PPARG2, FABP4 and FSP27 (10 μM CBD). ↑PPAR‐ γ2 (above 2.5 μM CBD). ↑ adipogenesis at concentrations above 5 μM in mouse cells and 2.5 μM in human cells. CBD does not increase the expression of the lipolytic genes PNPLA2 and LIPE.

Abbreviations: AD, adipogenesis; AD‐MSC, adipose‐derived mesenchymal stem cells; alloHCT, allogenic hematopoietic cell transplantation; ALS, amyothrophic lateral sclerosis; AMSC, adipose mesenchymal stem cell; APL, alkaline phosphatase; APSC, apical papilla stem cell; BM‐MSC, bone marrow mesenchymal stem cells; BRDU, bromodeoxyuridine; CBD, cannabidiol; CM, commercial; DFC, 2′,7'dichlorofluoresceindiacetate; DFSC, dental follicle stem cell; DNPH, 2,4‐dinitrophenylhydrazine; DPSC, dental pulp stem cell; GVHD, graft‐versus‐host disease; hGMSC, human gingivial mesenchymal stem cell; hiPSC‐CM, human induced pluripotent stem cell‐derived cardiomyocytes; HNP, hybrid nanoparticles; HO‐1, heme oxigenase‐1; hPLDSC, human peridontal ligament stem cells; LPS, lipopolysaccharides; MOR, moringin; OTG, osteogenesis; OTP, osteoprotection; OTR, osteoregeneratio; PLGA, poly (lactic‐co‐glycolic acid). PPAR‐γ, peroxisome proliferator‐activated receptor gamma; RS, reticulum stress; RXR‐α, retinoid X receptor alpha; SSPC, skeletal stem and progenitor cell; TBARS, triobarbituric acid‐reactive substances; TGF‐β, transforming growth factor beta.

**TABLE 2:** CBD and its protective effect on SSC.

Aim	Ref	Stem cell isolation	Methodology	Results
NP	Ivanov et al.^52^	Human embryonic neuronal cells obtained from Gibco/life technologies.	In vitro: Immunohistochemistry of the markers nestin to evaluate stemness and doublecortin to mark differentiation. Study of proapoptotic markers with ELISA. Study of gene expression and regulation by western blot assay, RT‐qPCR and evaluation of TNF‐cell promoter interaction by luciferase assay.	No negative effect was observed in the use of CBD in neural stem cells (5–15 μM). ↓ Sox2 (10 μM). Protective effect against 5 Gy radiation (effective against glioblastoma) at concentrations of 5–15 μM 0.5 h after irradiation.
	Hou et al.^53^	NE‐4C neuroectodermal mouse stem cell line. Purchased.	In vitro: Western blot assay and cell viability assay by MTT. In vivo: Male C57Bl/6J mice with depressive behavior. CBD administration at 10 mg/kg concentration. RT‐qPCR, RNA sequencing and transcriptome analysis for gene expression assessment.	In vitro: CBD reverse the negative effect of corticosterone on NE‐4C proliferation In vivo: ↓ depressive, ↓ serum corticosterone and ↓ animal's anxiety. Reverse abnormal neurogenesis in depressive mice.
	Razavi et al.^48^	‐	In vivo: Adult male albino Wistar rats. CBD dissolved in 10% DMSO and 90% phosphate buffer, injecting 5 μL of the solution into the lateral ventricle of the brain (50 μg CBD/5 μL). After the experiment, the sample was excised and microinjected, and a fluorescence immunohistochemistry assay was performed.	↑ cell proliferation in abstinence rats ↑ neuroblast by a ↑ neurogenesis in abstinence rats. ↓ neuronal degeneration generated by this period. ↓ number of dark neurons by CBD treatment in abstinence rats.
	Večeřa et al.^54^	D3 wild type mouse embryonic stem cells and HDAC1 mESC from the mouse blastocysts.	In vitro: Western Blot to detect proteins related to histone acetylation or deacetylation and cell differentiation. In vivo: C57/BL6 mice with schizophrenia. CBD was administered in doses of 10 mg/kg or 30 mg/kg by injection. Brain cryosectioning and immunocytochemistry with anti‐Sox2 and anti‐H3K9.	↓NCAM levels in the hippocampus, reversing the abnormal H3K9 deacetylation characteristic in schizophrenia. ↑the activation of the Sox2 protein, which means that acetylation levels have normalized recovering the stemness of the NSCs
	Rajan et al.^55^	hGMSC cells obtained from human gingival biopsies.	In vitro: Sequencing of an RNA library and RT‐qPCR.	↑ E2F1, NDUFC1, PARD3B, RAPH1, NDUFV3, SDHC, MAP1B, FKBP4; ↓ PPP3CC, TARBP2, HTRA2, STRADA, STRADB, TRAK2, CABINI, VDAC1. ↑ MAPKAP1, TRAP1, STK25, STIP1, HSPB1, HERPUD1, NFE2L2; ↓ MAPK12, MAPK14, MICAL1, MICAL2, MICAL3.
CP	Fouda et al.^56^	Hamster ovary cells and hiPSC‐CM. Purchased.	In vitro: Electrophysiological study of the cell membrane and the activation and inactivation of voltage‐dependent channels to simulate the action potential.	CBD protects cells from hyperexcitability and cytotoxicity by acting as a cardioprotective molecule at 5 μM doses. ↓INap, PMA, CPT‐cAMP and ADP Right‐shift the midpoint of activation V_½_ and ↓ z of activation curve.
	Franco‐vadillo et al.^57^	Use of hiPSC‐CM. Purchased.	In vitro: Oxidative stress assay and study of cell apoptosis by means of the evaluation of caspases 3/7 activity by luciferases. The viability of cardiomyocytes was determined with a cell extract of human macrophages previously treated with LPS.	Reversal of the negative electrophysiological effect exerted by LPS on the cell membrane. CBD reverse ROS levels after LPS or MPLA incubation (5 μM). = TNF‐α and IL‐6 after CBD‐dose in control cells. ↑TNF‐α and ↓ IL‐6 after CBD‐dose in LPS and MPLA‐stimulated cells (5 μM).
IP	Chiricosta et al.^7^	Human mesenchymal gingival stem cells from human biopsies	In vitro: Cytotoxic assay by MTT, morphological analysis of cells and Western Blot assays and RNA extraction.	↓ inflammation through activation of the TGF‐β pathway and inhibition of the IL‐1 and JAK/STAT pathways at a concentration of 5 μM. Modification in TNF‐α, IL‐1, IL‐6 and TGF‐β gene expression Apoptosis reduction by a caspase eight downregulation. ↓ NF‐kB
	Yeshurun et al.^58^	Clinical trial, phase II: *N* = 48 patients CBD dissolved in olive oil at 2.5% with an amount of 150 mg per dose, two doses per day. Study of chimerism in bone marrow by XY fluorescence in situ hybridization analysis, DNA amplification and histological study of some samples.		No toxic effect has been observed in the use of CBD. Treated patients have not developed acute GVHD during the 30 days of treatment. Disease development after CBD discontinuation in eight of the patients. No significant differences between CBD and historical control patients treated.
	Ruhl et al.^59^	AMSC obtained from human subcutaneous adipose biopsy samples.	In vitro: Analysis of metabolic activity using a PrestoBlue assay and cell viability assay by crystal violet. Numerous assays, such as growth factor assessment by ELISA, DNPH protein carbonylation assay, lipid peroxidation assessment by TBARS, Oil Red O staining assay or the chondrogenic tissue assay.	No IL‐6 reduction in LPS‐induced inflammation at low doses (3 μM). ↓ oxidative stress and inflammatory effect of LPS on stem cells. ↑ VEGF production of CBD in a LPS‐induced culture (↑ vascularization)
	Libro et al.^60^	hGMSC obtained from biopsies of healthy patients.	In vitro: Cytofluorimetry for the evaluation of cell markers. RNA library sequencing, RT‐qPCR and Western Blot to determine gene expression. Finally, immunohistochemistry was performed.	No morphological differences between normal hGMSC and CBD‐treated hGMSC. ↓ expression of genes related to NOD‐like, TNF, Jak–STAT and NF‐kB signaling pathways. ↑ immunitary response inhibitors antigens CD47, CD55, CD276.
ERSP	Kowalczuk et al.^61^	Human adipose‐derived stem cells HuASC from human biopsies	In vitro: Morphology study by confocal microscope. Cell proliferation assay with commercial kit and Ki‐67 immunostaining. RT‐qPCR and Western blot. Other studies such as evaluation of oxidative stress, nitric oxide, cell cycle analysis, migration by wound healing assay and B‐galactosidase activation.	↑ actine density, rounder nucleus and ↑ mitochondrial distribution than stressed and normal ASCs (5 μM). No proliferative or cytotoxicity effect of CBD in cells at 1, 5 and 10 μM. Cytotoxicity effect above 50 μM. ↑ proliferation in stressed cells with a CBD pre‐treatment (5 μM). ↓ cell apoptosis by a ↑ Superoxide dismutase 2, Glutathione peroxidase and SIRT1 (5 μM). ↓ TNF‐α, IL‐4, IL10, and ↑ IL‐1b (5 μM). ↑ IL‐6 mRNA but ↓ IL‐6 protein.
OSP	Bublitz et al.^62^	AD‐MSC from human subcutaneous adipose tissue.	In vitro: RT‐qPCR, Western Blot and cell metabolism test with WST‐1	↓ apoptosis and ↓ ROS by a ↑ HO‐1 protein and mRNA with 3 μM CBD dose (max. value after 48 h incubation) by a non‐receptor effect. ↑ metabolic activity after 24 h incubation with 1–3 μM CBD. slightly ↑ autophagy by ↑ LC3A/B‐II.
SE	Deng et al.^63^	NPCs obtained from the forebrain of mouse pups.	In vitro: Quantification of proliferation by ELISA and WST‐1.	↓ viability and proliferation of NPC cells and glioblastoma cells. CBD + TMZ = no cytotoxicity effect against NPCs (only cancer). CBD + BCNU = synergistic antiproliferative response (cancer and NPCs). CBD + BCNU = synergistic, additive and antagonist (cancer and NPCs). CBD + CDDP = no cytotoxic effect against NPCs (only cancer).
	Libro et al.^64^	hGMSC obtained from biopsies of healthy patients.	In vitro: Transcription study by deep sequencing of RNA libraries and RT‐qPCR. Immunocytochemistry.	↓ of kinase expression (5 μM): GSK3β, CDK5, DYRK1A, CAMK2A, MAPK1, MAPK12, MAPK14. Modulation of the hGMSC transcriptome, ↓ expression of genes involved in Alzheimer's pathogenesis. ↓ expression of GSK3b and p‐GSK303B2, which plays a key role in Alzheimer's disease.
SCR	Miller et al.^65^	BM‐MSC and ASC, both obtained from human and porcine biopsies.	In vitro: Cell migration and proliferation assay with MTT in human cells. Wound‐healing assay in porcine cells.	↑ migratory capacity in human BM‐MSCs and ASCs. Higher values of migration with low CBD concentration (300 nM) than high ones (3 μM). ↑ proliferation in BM‐MSCs with a greater effect at low doses (300 nM) than at high doses (3 μM). ↑ wound closure in porcine cells in the presence of CBD. Higher wound‐closure with low CBD concentration (300 nM).
DS	Sun et al.^66^	Cells obtained from the cerebral cortex of E18 Sprague–Dawley rat embryos. The iPSC cell line 6358–1 were obtained by reprogramming skin fibroblasts from patients with Dravet syndrome, performed by Stanford University.	In vitro: Neuronal calcium assay by FLIPR Tetra Cellular Screening System and evaluation of neuronal cell excitability.	Cytotoxicity effect with high CBD doses after 48 h (15 μM) Rat CNC: modulatory effect on the excitability at low concentrations (50–500 nM). iPSC: ↑ excitability of inhibitory neurons and ↓ excitability of excitatory neurons without altering the sodium amplitude of the cells 50 nM.

Abbreviations: AD‐MSC, adipose‐derived mesenchymal stem cells; alloHCT, allogenic hematopoietic cell transplantation; ALS, amyothrophic lateral sclerosis; AMSC, adipose mesenchymal stem cell; APD, AP duration; APL, alkaline phosphatase; APSC, apical papilla stem cell; BM‐MSC, bone marrow mesenchymal stem cells; BRDU, bromodeoxyuridine; CBD, cannabidiol; CBG, cannabigerol; CM, commercial; CP, cardioprotective; DFC, 2′,7'dichlorofluoresceindiacetate; DFSC, dental follicle stem cell; DNPH, 2,4‐dinitrophenylhydrazine; DPSC, dental pulp stem cell; DS, disease study; ERSP, endoplasmic reticulum stress protection; GVHD, graft‐versus‐host disease; hGMSC, human gingivial mesenchymal stem cell; hiPSC‐CM, human induced pluripotent stem cell‐derived cardiomyocytes; HO‐1, heme oxigenase‐1; hPLDSC, human peridontal ligament stem cells; LPS, lipopolysaccharides; MPLA, monophosphoryl lipid A; NP, neuroprotective; OSP, oxidative stress protection; PLGA, poly (lactic‐co‐glycolic acid); PPAR‐γ, peroxisome proliferator‐activated receptor gamma; rCNC, rat cortical neural cells; RS, reticulum stress; RXR‐α, receptor X retinoide alfa.

**TABLE 3:** CBD as an antitumor agent and its effect on CSCs.

Tumor	Ref	In vitro/in vivo	Treatment	Results
GBM	Lah et al.^12^	In vitro: GBM cell lines: U373 and 9 primary GBM cells from patients (NIB140, NIB142, NIB138, NIB180, NIB185, NIB182, NIB167, NIB258, NIB255) GBM stem cell (GSC) lines: NCH644 and 7 primary GSC from patients (K26, NIB216, NIB237, NIB225, NIB220, NIB249, NIB253)	Different concentrations (0.32–320 μM) of CBD alone or combined with CBG at the molar ratio CBD:CBG 3:1 (7.6:2.5–245:80 μM) with 100 μM temozolomide for 48 h.	IC_50_ Values in cells treated with CBD or CBD:CBG (3:1), respectively: GBM cell lines: Mean (78.9 ± 7.8; 76 ± 6.6:25 ± 2.1 μM) GSC lines: Mean (50 ± 7.1; 42 ± 3.4:16 ± 1.1 μM) NCH644 (34; 34:11 μM); K26 (73; 54:18 μM); NIB216 (24; 30:10 μM); NIB237 (50; 27:9 μM); NIB225 (50; 28:9 μM); NIB220 (84; 38:12 μM); NIB249 (52; 46:15 μM); NIB253 (35; 30:10 μM)
	Volmar et al.^67^	In vitro: Primary cell cultures of GSC from human GBM biopsies In vivo: B6.129S6‐Rag2^tm1Fwa^ N12 (immunodeficient Rag2‐KO) mice with intracranial orthotopic xenografts of GSC (NCH421K and Line2)	In vitro: CBD (10 μM) for 6 hours TMZ (100 and 300 μM) Control: 0.01% DMSO In vivo: Mice were i.p. injected every other day for 21 days with CBD (15 mg/kg) or vehicle (5% Tween 80, 5% ethanol in 0.9% saline).	In vitro CBD treatment: Differentially affected the GSC panel, dividing cells into CBD‐sensitive (Line7, NCH592B, GBM20, NCH684, GBM29, Line6, NCH644, Line2, NCH588J, GBM10, Line11, BT423, BT112) and CBD‐insensitive (NCH421K, NCH441, Line9, Line8, GBM14, Line10, GBM13, BT172). ↑ cytotoxicity than TMZ in CBD‐sensitive GSC. In vivo CBD treatment: ↑ survival of mice inoculated with Line2 GSC, but no response in animals bearing NCH421K GSC.
	Lah et al.^68^	In vitro: GSCs lines (established lines NCH644, NCH421k, and patient‐derived K26)	Different concentrations (0.16–50 μM) of CBG, CBD, and THC alone or combined with TMZ (100 μM or 400 μM) for 48 h	IC_50_ Values CBD: NCH644: 15.9 μM; NCH421k: 27.9 μM; K26: 14.6 μM; Mean in GSCs: 19.5 ± 4.2 μM CBG: Mean in GSCs: 59.0 ± 14.7 μM THC: Mean in GSCs: 22.8 ± 3.3 μM
	López‐Valero et al.^69^	In vitro: Glioblastoma patient‐derived GICs (GH2‐GICs and 12O12‐GICs cells) In vivo: Orthotopic xenografts 7.5 × 10^4^ 12O12 GICs (*n* = 6–7) generated by intracranial injection into the right cerebral hemisphere of immunodeficient nude mice	In vitro (for 5 days): Vehicle (DMSO) THC:CBD (1:1 ratio) [2.5 μM THC + 2.5 μM CBD] THC:CBD (1:5 ratio) [0.83 μM THC + 4.17 μM CBD] TMZ (100 μM in GH2‐GICs and 20 μM in 12O12‐GICs); In vivo (oral admin for 4 weeks): THC:CBD (1:1 ratio) [THC (5 mg/kg) + CBD (5 mg/kg)] THC:CBD (1:5 ratio) [THC (5 mg/kg) + CBD (25 mg/kg)] TMZ (5 mg/kg, I.P. administration) (THC and CBD diluted in sesame oil)	In vitro: ↓ proliferation: GH2‐GICs: THC:CBD (1:5), or THC:CBD (1:1 or 1:5) + TMZ > THC:CBD (1:1) or TMZ alone 12O12‐GICs: similar results with the strongest effect with THC:CBD 1:5 + TMZ ↓ neurosphere formation and apoptosis: THC:CBD (1:5) > TMZ or THC:CBD (1:1) THC:CBD (1:5) + TMZ > THC:CBD (1:1) + TMZ In vivo: ↓ tumor growth and ↑ survival of the animals: THC:CBD (1:5) + TMZ > THC:CBD (1:1) + TMZ > TMZ THC:CBD (1:5): no effect on tumor size but ↑ survival of the animals
	Nabissi et al.^70^	In vitro: GSC lines (#1, #30 and #83) isolated from patients	Cell viability: CBD (from 0.5 to 50 μM) for 24 h. Rest of experiments: 10 μM CBD for up to 24 hr	CBD induced activation of autophagy via TRPV2 pathway ↓ viability and proliferation, with IC_50_ 24 h: 19.4 μM (#1); 14.6 μM (#30); and 19.3 μM (#83) Cell cycle arrest at G_0_/G_1_ phase; ↓ neurosphere formation; ↓ stem cell markers.
	Singer et al.^71^	In vitro: Primary GSC lines (3832 and 387) In vivo: Two models of intracranial GBM xenografts in female athymic nu/nu mice, established by intracranial injection of a low number (5 × 10^3^) of luciferase‐labeled GSC lines 3832 and 387	In vitro: Cell viability assay: Range of CBD concentrations for 48 h Rest of experiments: CBD (2 μM) alone or with 40 μM antioxidant VitE for 48 h In vivo: CBD (intraperitoneally 15 mg/kg, 5 days per week)	In vitro CBD treatment: ↓ viability of 3832 and 387 GSCs (IC_50_: 3.5 and 2.6 μM, respectively). ↓ stemness marker and ↑ MES markers. In vivo CBD treatment: ↑ survival in tumor‐bearing mice ↓ p‐AKT, Ki67 and ↑ caspase‐3 activation in GBM xenograft tissues ↓ tumor size (at day 22), but tumors became resistant to treatment 1 week later (day 29)
	Soroceanu et al.^72^	In vitro: Neurospheres derived from GBM primary cells from patients	Neurosphere formation assay: 1 μM CBD	CBD: ↓ neurosphere formation (reduction of ~60%) ↓ expression levels of Id‐1 and SOX2 in neurospheres
Lung	Salles et al.^73^	In vivo: 2 groups (*N* = 10/group) of male nude mice with a subcutaneous tumor into the flank (5 x 10^5^ NCI‐H1437)	Inhalant CBD and placebo (hemp seed oil). Each animal received 6.4 mg of CBD per day (four sprays of 1.6 mg of CBD/mouse/day) for 3 weeks	CBD vs. placebo: ↓ tumor growth rate ↓ CD44 expression (from 12.1 to 3.2% of total tumor cells) by flow cytometry analysis and by immunohistochemistry
	Misri et al.^74^	In vitro: NSCLC‐CR cell lines (H460‐CR and A549‐CR) CSC spheres derived from H460‐CR and A549‐CR.	H460‐CR and A549‐CR: increasing concentrations of cisplatin and CBD for 48 h. Forming CSC spheres: CBD (15.8 μM), or cisplatin (27 μM) during sphere formation for 7–10 days.	CBD: ↓ spheres formation in treated NSCLC‐CR vs. control or cisplatin‐treated cells. ↓ CD44+ and CD133+ cell population in treated spheres (41.73% in H460‐CR, and 81.70% in A549‐CR;) vs. controls (91% in H460, and 95.877% in A549) ↓ protein expression of Snail, Nanog, and Vimentin in both NSCLC‐CR
	Hamad et al.^75^	In vitro: NSCLC cell lines (A549 and H1299) SCLC cell line (H69) Spheres derived from A549, H1299 and H69	Viability assay: CBD concentrations (0–48 μM) for 24 h Sphere formation: Preincubation with 10 μM CBD for 24 h in the presence or absence of serum Rest of assays: Spheres were incubated with 10 μM CBD for 24 h.	CBD vs. control: ↓ viability of NSCLC cell and spheres with different sensibility. ↓ sphere formation. ↓ expression levels of: SOX2, Oct‐4 and CD133 (in A549 spheres); SOX2 and CD133 (in H1299 spheres) and SOX2, CD44, CD133 and Oct‐4 (in H69 spheres). ↑ annexin‐positive cells in A549 spheres (~30%), in H1299 spheres (10%–15%) and in H69 spheres. ↑ of caspase 3/7 in A549, H1299 and H69 spheres. ↑ expression of TP53 (3‐fold), CDKN1A (5‐fold) and pro‐apoptotic genes BAK1, BAX, and BAD (~1.8‐fold) in A549.
Breast	Jo et al.^76^	In vitro: Mammosphere derived from MCF7	CBD (2 μM) for 2 weeks	CBD: ↓ formation and growth of mammospheres. ↓ expression levels of stem cell proteins (ALDH1A1, CD133, NANOG, SOX2) in treated mammospheres vs. untreated.

Abbreviations: Aml‐1, acute myeloid leukemia; BCNU, carmustine; CBD, cannabidiol; CR, cisplatin‐resistant; CSC, cancer stem cell; DMSO, dimethylsulfoxide; ERA, erastin; GBM, glioblastoma multiforme; GICs, glioma initiating cells; GSC, glioblastoma stem cells; IC_50_, half‐maximal inhibitory concentration; i.p, intraperitoneal; KO, knock‐out; MES, mesenchymal; NSCLC, non‐small‐cell lung cancer; PE, piperazine erastine; PN, proneural; ROS, reactive oxygen species; SAS, sulfasalazine; THC, tetrahidrocannabinol; TMZ, temozolomide.

## 3 RESULTS AND DISCUSSION

**FIGURE 2:**
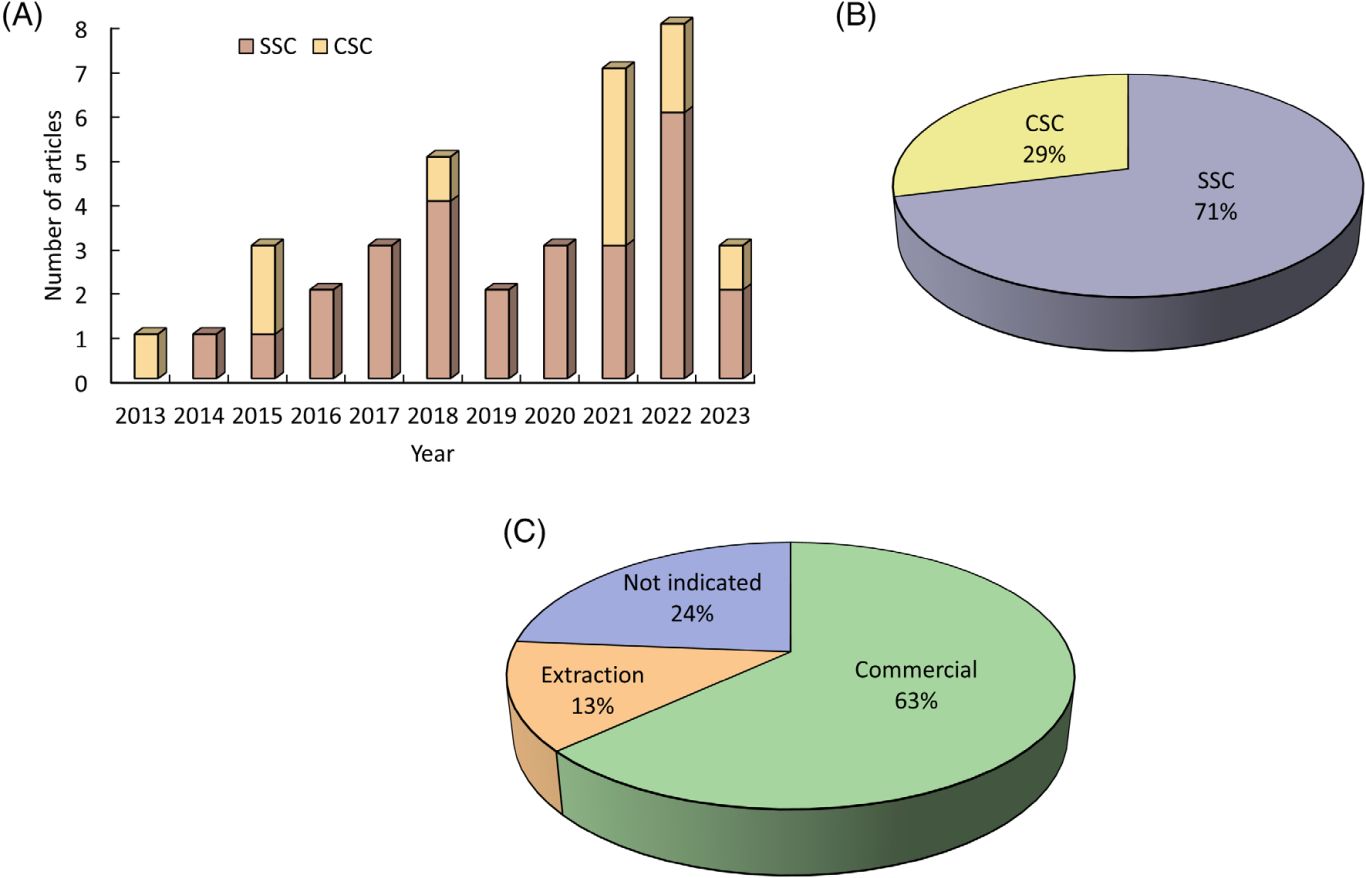
Manuscripts studying the effect of CBD on SSC or CSC over time. Graphical representation of: (A) Number of articles published; (B) Percentage of articles analyzing SSC and CCS; (C) Percentage of articles that use commercial CBD or plant extract.

The analysis of the 38 selected articles showed that CBD assays in stem cells increased in the past 10 years, with 2015, 2021, and 2022 standing out in relation to CSC studies. Assays in SCC also progressively increased during this time (Figure 2A). Most of the manuscripts focused on the effect of CBD on SSC (71%) and only a 29% on CSC (Figure 2B). These studies used commercial CBD (63%) and CBD from plant extractions (13%). No specific origin was indicated in 24% of the manuscripts (Figure 2C).

### 3.1 CBD enhance stem cell differentiation

Eleven of the selected articles analyzed the effect of CBD on stem cell differentiation (Table 1). Three manuscript used MSCs as a cellular model for the study of osteogenesis,^41^, ^42^, ^44^ two manuscripts studied osteogenesis in tooth stem cells, specifically apical papilla stem cells (APSCs), dental follicle stem cells (DFSCs) and dental pulp stem cells (DPSCs)^43^, ^45^ and only one used skeletal stem and progenitor cells (SSPC).^46^ CBD induced a direct modulation of the transcriptome and gene expression, enhancing the expression of osteogenic genes such as the BMP family^44^ *Runx2*,^42^, ^45^, ^46^ or *ALP*.^44^, ^45^ CBD was also able to modulate some signaling pathways for cell protection, such as the p42/44 MAPK^44^ or p38 MAPK.^42^

Yu et al.^45^ and Petrescu et al.^43^ studied osteogenesis in tooth stem cells, specifically apical papilla stem cells (APSCs), dental follicle stem cells (DFSCs) and dental pulp stem cells (DPSCs). CBD (2.5 and 12.5 μM) increased cell proliferation and protection against inflammation in DPSC and modulated osteogenic gene expression such as *ALP*, osteoporin, osteocalcin, osteonectin, or type 1 collagen. APSC also increased osteogenic gene expression and DFSC shows a mineralization increase. Finally, Ihejirika‐Lomedico et al.^46^ were the only ones to perform an osteo‐genic study with SSPC, in which increased expression in the osteogenic genes *Runx2* and *Osterix* was observed. Interestingly, these authors demonstrated that CBD administered via osmotic pump in mice with bone fractures or osteoporosis (induced by ovarectomy or fluoxetine) significantly improved bone regeneration.

In addition, in vivo studies by Kamali et al.^41^ in mice with bone injury showed an increase in scaffold regenerative capacity in presence of CBD due to an enhancement in the MSC and an osteogenic effect because of an upregulation in the osteogenesis genes osteoporin, osteocalcin, and osteonectin and an increased CB1 and CB2 in DPSC cells. Also, CBD reduced the effect of TNF‐α on the migration of DPSC at 2, 5 μM concentration.

Nowadays, osteostimulation of endogenous stem cells is the main therapy for bone repair and osteoporosis. There are numerous ways to stimulate the differentiation, recruitment and proliferation of these stem cells, such as electrical stimulation,^77^ physical support,^78^ or drug treatment^79^, ^80^, ^81^, ^82^, ^83^ which include the endocannabinoids. Most of the articles in this review on osteoregeneration use only the drug except the experiment by Kamali et al. in which this drug enhance the regenerative effect of the scaffold.^41^

Regarding neurogenesis, various authors have demonstrated CBD's ability to enhance stem cell differentiation in the nervous system and reduce inflammation and degeneration processes.^47^, ^48^, ^49^ Cellular exposure to a siRNA and CBD liposome‐coated nanoparticle increased the cell viability, reduced inflammation, and downregulated the htt gene expression, mitigating the effects of Huntington's disease.^47^ Additionally, CBD promoted SSC survival and neuronal differentiation through PI3K/Akt/mTOR activation, further enhanced by the presence of Moringin.^49^ Razavi et al.^48^ conducted an in vivo study using rats previously administered with methamphetamine, showing that CBD treatment increased neurogenesis in the hippocampus and reduced neuronal degeneration. In neuropathy, there are evidences of the positive effect of NSC engraftment as a treatment. However, NSC cells in the adult generate a reduced number of neurons, so they must be accompanied by molecules that enhance neurogenesis.^84^

Finally, Fellous et al.^50^ and Chang et al.^51^ investigated adipogenesis in human and mouse BM‐MSCs, as well as human AD‐MSCs. The use of CBD induced PPAR‐y activation, a protective pro‐differentiator and an antilipolytic effect by modulating the expression of genes such as *PNPLA2*, *LIPE* or adiponectin. Fellous et al.^50^ evidenced an increased fibroblast colony formation of MSC and upregulation of *Fabp4*, *Glut* and *Scd* genes with a 5 μM CBD concentration. On the other hand, Chang et al.^51^ shown an increased lipid accumulation and adipogenesis in mouse and human MSC in a concentration above 5 and 2, 5 μM respectively. The interaction of CBD with PPAR‐γ have an important role in the adipogenesis, developing insulin‐resistance and lipodystrophy in knockout PPAR‐γ MSC cells.^85^

### 3.2 CBD and its protective effect on SSC

**FIGURE 3:**
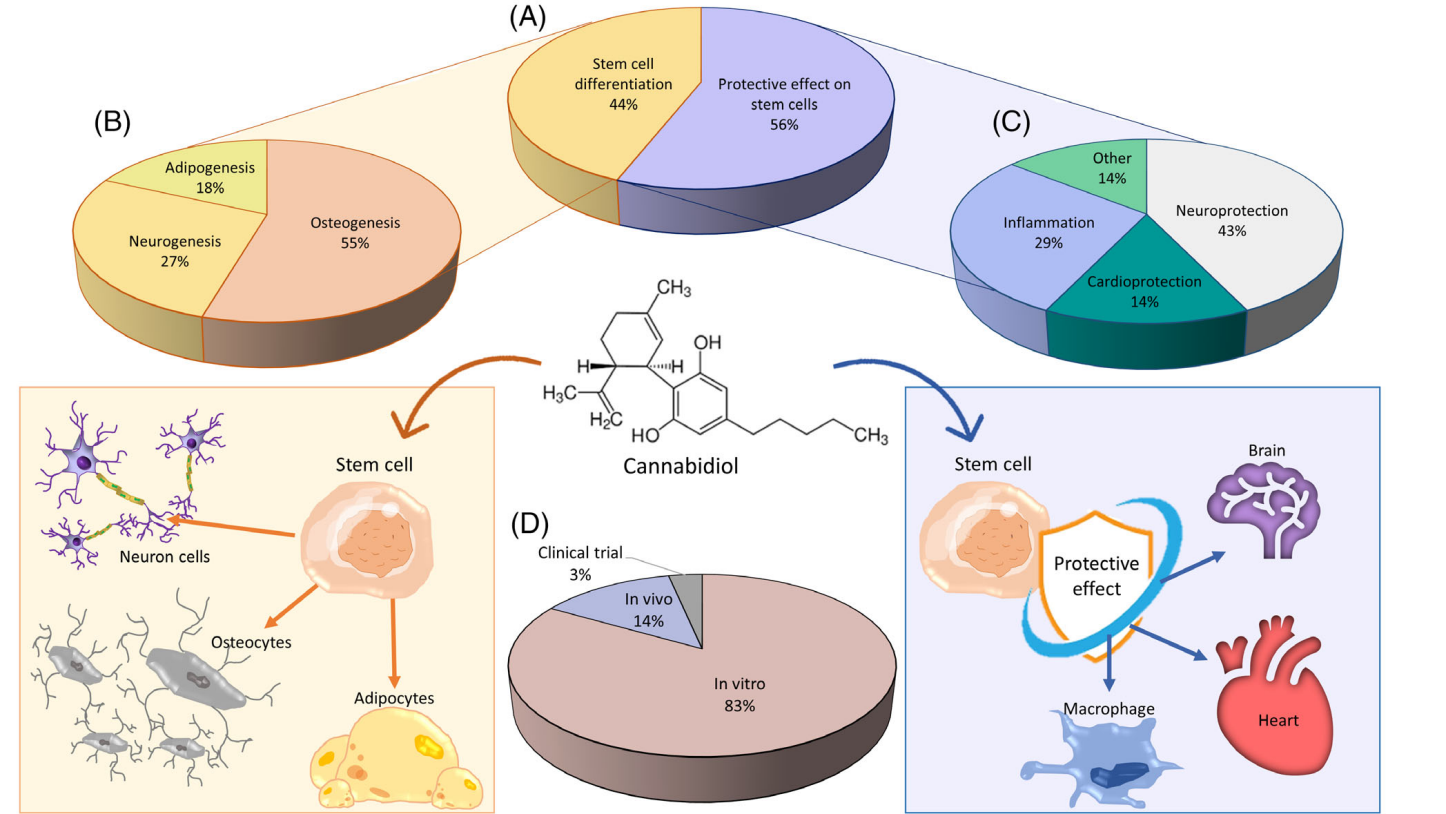
CBD acts as a differentiator inducer in stem cells and has a protective effect on them. Graphical representation of: (A) Percentage of articles studying organogenesis and protective effect; (B) Percentage of studies on adipogenesis, neurogenesis and osteogenesis; (C) Percentage of manuscripts studying neuroprotection, cardioprotection and inflammation among others; (D) Percentage of studies carrying out in vitro, in vivo and clinical trials.

Seventeen of the collected manuscripts studied the protective capacity of CBD on SSC (Table 2) (Figure 3), one of them present in both Tables 1 and 2.^48^ CBD was safe against human embryonic neural stem cells and also protected them after a radiation exposure over 5 Gy (radiation dose effective against glioblastoma) in an interval of 5 to 15 μM with a Sox2 reduction.^52^ Protective effect against radiation has been previously studied with a positive effect, for example, in keratinocytes and melanocytes cultures in CBD concentration of 8 and 4 μM respectively, probably by an anti‐inflammatory and antioxidative effect due to a CB1 and CB2 activation.^86^, ^87^

The study of Hou et al.^53^ shown an upregulation in FoxO pathway, reducing the impact of corticosterone on an in vitro model of neuroectodermal mouse stem cells.^53^ Also, CBD reduced the corticosterone production and effect on abnormal neurogenesis, anxiety and depressive behavior on CBD‐treated depressed‐like mice. Other study shows a reversal of H3K9 deacetylation by a NCAM level reduction and an enhanced acetylation by a Sox2 activation in schizophrenia mice models after the CBD injection.^54^ The NCAM adhesion molecule response can be developed by a CB1 receptor activation, enhancing the neurogenesis and differentiation by a CBD treatment.^88^

Another disease studied was the Amyotrophic Lateral Sclerosis (ALS), where CBD reduced its development due to a modification of the expression of some genes related to the disease, such excitotoxicity‐related, oxidative stress‐related, or mitochondrial dysfunction‐related genes at a 5 μM concentration.^55^ Fouda and Franco‐Vadillo performed the only two manuscripts related to cardioprotection present in this systematic review, using induced human stem cells (hiPSCs) in both of them. Their first study shows a cardioprotective effect of CBD at 5 μM concentrations in a pro‐inflammatory environment and a high glucose‐induced arrhythmia reversal effect due to a INap, PMA, CTP‐cAMP, and ADP downregulation, leading a correct cell differentiation under adverse situations. A year later, they studied the protective capacity of the drug against an LPS and MPLA induced inflammation. At 5 μM concentration, CBD reversed the abnormal electrophysiology, ADP increment, and caspase‐3 activity in the LPS and MPLA‐induced cells. Also, CBD induced a reduction in IL‐6 and activation in TNF‐α only in LPS and MPLA‐induced cells.^56^ CBD can activate RISK pathway and increase the AKT protein production and its phosphorylation into p‐AKT, producing a beneficial effect in the myocardial reperfusion damage treatment.^57^ Also, CBD can be used as anti‐inflammatory and antioxidative molecules against a cardiomyopathy due to a reduction in cellular damaging and caspase‐related apoptosis, a mitochondrial protection and a ionic homeostasis regulation.^89^

Four of the articles were aimed only in the anti‐inflammatory effect (Figure 3). The research team of Chiricosta et al.^90^ spotted a reduction in the inflammatory response in human mesenchymal gingivial stem cells (5 μM CBD), despite of an increased TNF‐α. This anti‐inflammatory effect depends on some gene expression modifications that allowed an increased TGF‐β expression and an inhibition of IL‐1 and downregulation of the pro‐inflammatoriy pathway JAK/STAT (and then reducing the IL‐6 production). Also, a reduction in NF‐kB production and a caspase 8 downregulation have been shown.^60^ CBD also performed a reduction on the oxidative stress and inflammatory environment effects against adipogenesis without a IL‐6 reduction and an increased VEGF production in AMSC with low CBD concentration (3 μM).^59^ The treatment of hGMSC with CBD reduced the apoptotic activation in the inflammated SSCs by an inhibition and downregulation of some pro‐apoptotic genes, and upregulation of Bcl‐2 and PI3K/Akt proteins. In addition, an anti‐inflammatory effect has been shown due to an inhibition of the NALP3‐inflammasome complex by a CB1 receptor activation. CBD also reduced the inmune response with a downregulation in genes related to HLA‐H, GNG1O, NLR, TNF, Jak–stat, and NF‐kB pathways, reduction in macrophage and T‐cell surface activators and increased anti‐immune response. An increased CD13, CD29, CD73 and reduced CD44, CD90 and CD199 could lead an increased adipogenesis against a pro‐inflammatory effect.^59^ One of the most studied CBD properties is the anti‐inflammatory effect, so its effects in the inflammatory pathways are well known. For example, CBD can reduce the NF‐κB activity in RAW 264.7 macrophages, probably by a p38 MAP kinase activity.^91^ Also, CBD can be a FKBP5 antagonist, reducing the NF‐κB activation.^92^ PPAR‐γ receptors also reduce the inflammatory environment reducing the NO, IL‐1β, IL‐6, TNF‐α, iNOS, and COX‐2 pro‐inflammatory influence by a NF‐Κb inhibition.^85^ Treatment with CBD can also inhibit IL‐2 and IFN‐γ expression at 0.5–20 μM concentration, two important JAK/STAT pathway activators.^93^

The study carried out by Yeshurun et al.^58^ was the only clinical study on CBD treatment in SSC. This study aimed to determine the capacity of CBD as a treatment for Graft‐versus‐Host disease after allogeneic implantation of hematopoietic stem cells. The study involved 48 patients, 28 of them received a donation from their HLA‐identical sibling, 16 received a donation from an unrelated but 10/10 matched individual and four of them received a donation from an individual with a single antigen mismatch. The doses used in this study were 300 mg of CBD each day for 30 days, divided into two daily doses of 150 mg intakes. The drug was administered orally, dissolved in olive oil at a ratio of 2.5%. The results of this study were positive, experiencing a significant reduction in the incidence of rejection during the 30 days of the experiment with no side effects observed, increasing the subsequent development of the disease after the elimination of CBD. Therefore, CBD has great potential in the treatment of this disease and can be added along with standard treatment to obtain better results.

Some of these studies carried out other pathways of protection of the SSC. One example is the protection against oxidative stress, where CBD (3 μM) reduced apoptosis and ROS due to an increased HO‐1 expression. Also, CBD increased AD‐MSC metabolic activity with a 1–3 μM.^62^ The increase of HO‐1 by CBD is also shown in the endothelial cells HUVEC by an independent TRPV1, CB1 and CB2 pathway, but by a N‐acetyl‐L‐cysteine (NAC) influence.^94^ This study also confirms an increased HUVEC metabolic activity in a 6 μM CBD concentration.

Endoplasmic reticulum stress is also a risk factor against SSC differentiation, and CBD shown a recovery of huASC proliferation and migration as well as a reduction of apoptosis.^61^ Treated SSC with 5 μM CBD shown an increased actine density and mitochondrial distribution with a rounder nucleus and an enhanced metabolism. CBD also increased the proliferation only in the stressed cells. CBD‐treated cells had a similar early apoptosis than untreated cells, but total cell apoptosis decreased by a Superoxide dismutase 2, Glutathione peroxidase and SIRT1 upregulation and P53, BAX and Cas‐9 downregulation. Also, CBD decreased the inflammatory response by a reduction in IL‐6 protein production, TNF‐α, IL‐4, and IL‐10 downregulation and IL‐1b upregulation. Lastly, CBD modified other cell factors like PERK, elF2‐α, CHOP, IRE, XBP1, etc.^61^ The study of Patel et al.^95^ shown an enhancement in the viability of mouse striatal cells with a CBD pre‐treatment by an increased GRP78 chaperone and ER‐resident neurotrophic factor MANF, and a reduction of BIM and caspase‐12 pro‐apoptotic molecules.

Two articles used non‐stem cell models as principal CBD target, but also studied it effect on SSC to discard any possible side effect. Deng et al.^63^ studied the antitumor effect of CBD in glioblastoma, so they also determined its side effect in NPCs, experiencing a reduction in their viability at the concentration used against glioblastoma. A CBD‐only treatment reduced viability and proliferation in NPCs, but CBD + TMZ and CBD + CDDP co‐treatments eliminated this drawback without influencing the antitumoral effect. On the other hand, Libro et al.^64^ used CBD against Alzheimer's disease at 5 μM, that leaded a modification in the expression of some genes such a reduction in secretases and kinases, increased ubiquitin‐conjugating enzymes and heat‐shock proteins and upregulation of PI3K/Akt protein production. Moreover, CBD reduced the expression of Alzheimer's disease‐related proteins by a TRPV receptor activation, such GSK3b. There are also evidences about the capacity of CBD to interact with glutamate receptors (NMDA receptors), propionic acid receptors (AMPA receptor) and PPAR‐γ, reducing the amyloid‐β production.^96^

The study of Miller et al.^65^ shown a proliferation and migration capacity of SSCs in the presence of CBD at concentration of 300 nM compared to concentration of 3 μM in human and positive wound‐healing effect in porcine BM‐MSC and ASC. Finally, Sun and Dolmetsch^66^ studied the use of CBD as a therapy against Dravet syndrome using iPSCs as a model. Concentrations of 50 μM modulated the excitability of inhibitory cells and reduced the excitability of excitatory neurons, and also shown a cytotoxicity effect with CBD after 48 h above 15 μM. Previous studies show the CBD potential as a Dravet syndrome treatment in pediatric clinical trials, enhancing the patient's quality of life and reducing the seizure frequency.^97^ When the long‐term efficacy and safety of CBD was examined in children and adults with treatment‐resistant epilepsy, specifically Lennox–Gastaut syndrome and Dravet syndrome, results indicate a median reduction of 50% in major motor seizures and 44% in total seizures after 12 weeks of CBD treatment. These effects were maintained for up to 96 weeks, with an acceptable safety profile. Common side effects included somnolence and diarrhea, supporting CBD's potential as a long‐term treatment option for Lennox–Gastaut syndrome and Dravet syndrome.^98^

On the other hand, Peters et al.^99^ explored the impact of cannabidiol (CBD) on the safety and effectiveness of diazepam nasal spray for seizure clusters. Their results indicated that CBD, especially in its FDA‐approved, highly purified form, did not compromise diazepam's safety or effectiveness. Patients used purified CBD required fewer secondary doses of diazepam compared to those used other or no CBD, with reduced treatment‐related adverse events, particularly in patients also taking clobazam. This supports safe concurrent use of CBD and diazepam nasal spray for appropriate epilepsy cases.

### 3.3 The effect of CBD on cancer stem cells

**FIGURE 4:**
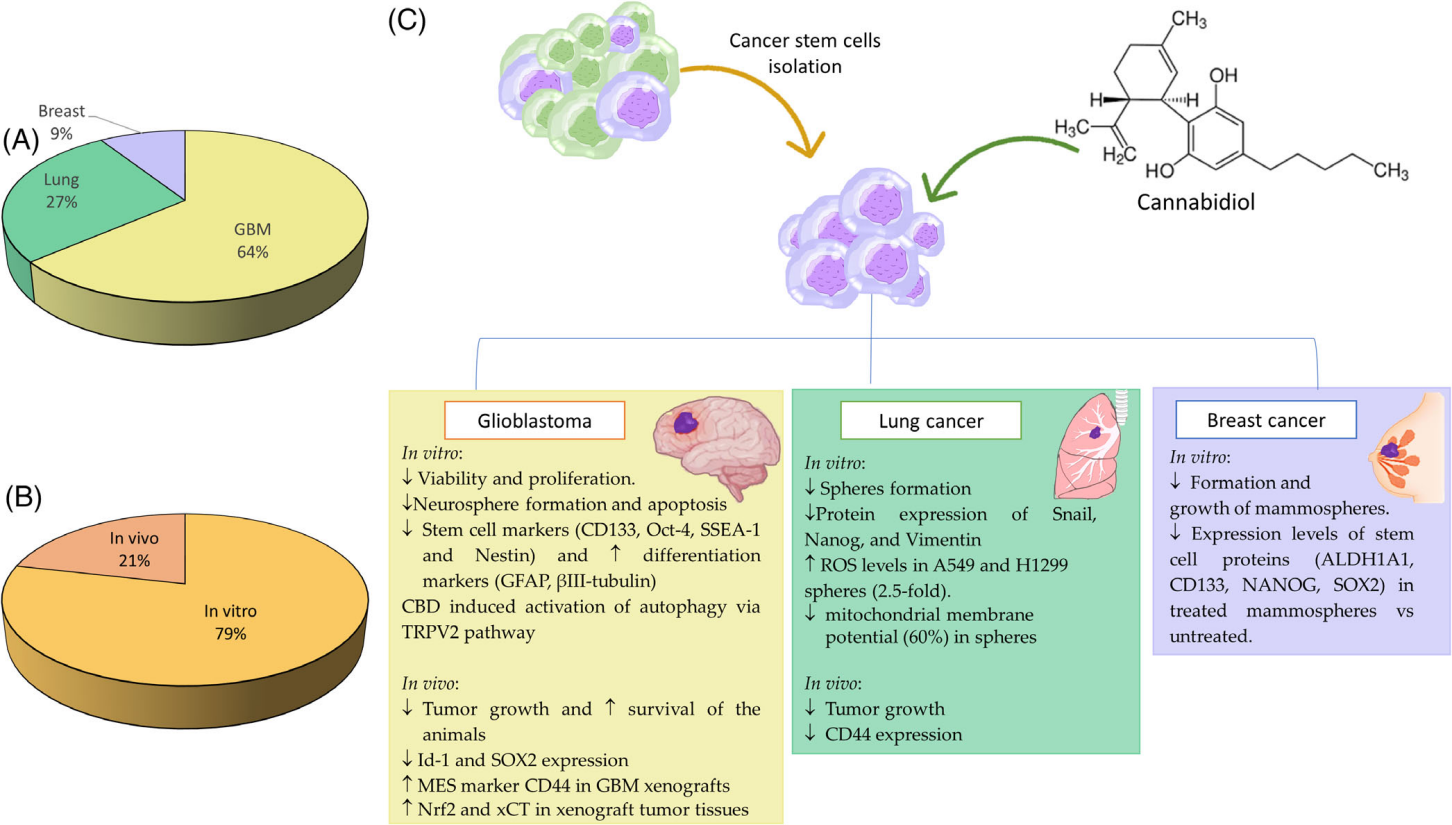
CBD presents antitumor activity against different types of aggressive tumors both in vitro and in vivo. Graphic representation of: (A) Types of cancer studied; (B) Percentage of in vitro and in vivo studies; (C) Main results of the effect of CBD on different types of CSC.

Eleven articles evaluated the effect of CBD specifically against CSCs of different tumor types (Figure 4). Seven articles studied the effect of CBD on glioblastoma. From them, most studies used GBM stem cells (GSCs) from established lines (e.g., NCH644 or NCH421k) or from primary cultures derived from patient samples. Three of the articles also performed in vivo assays to test the activity of CBD against intracranial GSC‐derived tumors.^67^, ^69^, ^71^ In addition, several studies have evaluated the impact of CBD on the formation of neurospheres from GBM patient cells. For instance, Soroceanu et al.^72^ reported a 60% reduction in neurosphere frequency after CBD treatment compared to untreated cells. Additionally, they showed that CBD decreased the expression levels of Id‐1 and SOX2 in the neurospheres formed.^72^ Id‐1 is a critical transcriptional regulator of glioblastoma aggressiveness and a marker of cancer stemness, making it a promising new drug target.^72^, ^100^

Volmar et al.^67^ examined CBD sensitivity in a panel of 21 human GSC lines. They classified the cells as CBD‐sensitive (Line7, NCH592B, GBM20, NCH684, GBM29, Line6, NCH644, Line2, NCH588J, GBM10, Line11, BT423 and BT112) and CBD‐insensitive (NCH421K, NCH441, Line9, Line8, GBM14, Line10, GBM13 and BT172). CBD also showed higher cytotoxicity than TMZ in CBD‐sensitive GSCs.^67^ These in vitro results were confirmed in vivo using immunodeficient Rag2 KO mice with orthotopic xenografts of GSCs (NCH421K and Line2). CBD administration prolonged the survival of mice inoculated with Line2, classified as a CBD‐sensitive GSC line, but showed no response in animals bearing NCH421K, a CBD‐insensitive GSC line. Regarding the mechanism of antitumor action, it was described that CBD effect was mediated by apoptosis or endoplasmic reticulum stress and autophagy in GSCs. In addition, CBD‐sensitive GSC death was associated with attenuation of canonical NF‐κB responsiveness, caused by nuclear accumulation and DNA binding of the RelA‐deficient phospho‐Ser311 subunit of NF‐κB. These results were complemented by transcriptomic analysis, which showed the repression of pathologically relevant genes of the RelA pathway, and by the application of a RelA inhibitor (SN50), which prevented the nuclear accumulation of RelA and blocked the cytotoxicity of CBD in Line 2, Line 11 and NCH644. In fact, CBD did not induce nuclear accumulation of RelA in CBD‐insensitive GSCs. On the other hand, CBD sensitivity was inversely correlated with ROS expression in GSCs both in vitro and in vivo, such that CBD‐sensitive cells expressed low levels of ROS, whereas CBD‐insensitive GSCs had high levels of ROS, so that ROS levels could be used as a biomarker for stratifying patients according to responsiveness to CBD treatment.^67^

The role of ROS was also evaluated by Singer et al.^71^ but in this case, they suggested that the cytotoxic effect of CBD in two primary GSC lines, 3832 (IC_50_: 3.5 μM) and 387 (IC_50_: 2.6 μM), was dependent on ROS action, as it was reversed by the addition of the antioxidant compound vitamin E (vitE). The authors reported that CBD inhibited GSC self‐renewal by increasing ROS levels, as evidenced by a decrease in sphere‐forming capacity. This effect was accompanied by activation of the p‐p38 pathway, downregulation of master regulators of self‐renewal (p‐STAT3, SOX2, and Id‐1), stemness markers (OLIG2), and proneural (PN) markers, and upregulation of mesenchymal (MES) markers (CD44, TNSFR10, CEBPB) in 3832 GSCs.^71^ The MES subtype in GBM has been associated with increased aggressiveness, poor patient outcomes, and recurrence of the tumor. In GBM, PMT may be equivalent to epithelial‐mesenchymal transition, which is associated with other aggressive cancers and is often responsible for resistance to radiation and chemotherapy.^101^

The study found that CBD treatment resulted in increased levels of the NRF2 targets SLC7A11/xCT and HMOX‐1, as well as an increase in the nuclear fraction of NRF2, indicating that CBD activates NRF2. Furthermore, inhibiting SLC7A11/xCT genetically or with drugs such as erastin (ERA) and piperazine erastine (PE) enhanced the cytotoxic activity of CBD and increased ROS levels in GSCs. In this study, the effects of CBD were also tested on two intracranial xenograft models of GSC lines 3832 and 387 in immunosuppressed mice. The results showed a significant improvement in survival rates, along with inhibition of p‐AKT, Ki67, and caspase‐3 activation in xenograft tissues. Similar to the in vitro results, CBD administration inhibited the expression of Id‐1 and SOX2, and upregulated CD44 and NRF2. Although the tumor volume was reduced in treated mice until day 22, the tumors became resistant after one week (day 29).^71^ CBD has previously been described to upregulate Nrf2, a key regulator of the cellular antioxidant system associated with neuroprotection.^102^, ^103^ It has been reported that NRF2 signaling is activated in CSCs and plays a significant role in tumor initiation, metastasis, and therapy resistance.^104^ In literature, Nrf2 has been shown to play a significant role in ROS‐mediated chemoresistance,^105^ as reported by Singer et al.^71^ These mechanisms could also explain the resistance by CBD‐insensitive GSC lines described by Volmar et al.^67^ which express high levels of ROS and likely have increased expression of cellular antioxidant mechanisms, such as Nrf2, that decrease the ROS production‐dependent cytotoxic effect of CBD. Furthermore, Volmar et al.^67^ proposed that co‐administration of ROS quenching agents could increase the sensitivity of CBD‐insensitive GSCs. However, Singer et al.^71^ contradicted this proposal by demonstrating that the cytotoxic effect of CBD was ROS‐dependent and was reversed by the addition of the antioxidant compound vitE. They proposed co‐administration of BBB‐permeable inhibitors of antioxidant mechanisms like the xCT system, as a strategy to reverse this resistance and enhance the effect of CBD.^71^ Therefore, further studies are needed to determine the role of ROS in CBD action against GSC.

Autophagy has been described as another mechanism of antitumor activity of CBD. Nabissi et al.^70^ described the induction of autophagy in GSC lines by CBD treatment, and detailed the molecular pathways involved in CBD‐driven autophagy and other molecular mechanisms triggered by its activation. They demonstrated that CBD‐induced autophagy was mediated by the TRPV2 signaling pathway and described the activation of autophagy genes, the increased expression of autophagy markers including LC3‐II and Beclin‐1/PI3K‐III complexes. They found that autophagy was also regulated by the AKT‐PI3k/RPS6KBI/PTEN pathway. Furthermore, CBD induces inhibition of viability and proliferation via activation of autophagy in GSC, with IC_50_ 24 h: 19.4 μM (#1); 14.6 μM (#30); and 19.3 μM (#83); cell cycle arrest at the G_0_/G_1_ phase and significant reduction of neurosphere formation. CBD‐induced autophagy has been associated with the promotion of Aml‐1a‐dependent GSCs differentiation, as it significantly decreased stem cell markers (CD133, Oct‐4, SSEA‐1 and Nestin) and increased levels of differentiation markers. CBD‐induced TRPV2‐dependent autophagy promoted the differentiation of GSCs and reduced their resistance to the cytotoxic effects of BCNU.^70^ Studies have shown that TRPV2 expression is lower in high‐grade gliomas compared to benign tissues. Additionally, increasing TRPV2 expression in low TRPV2‐expressing glioma cells has been found to inhibit cell survival and induce apoptosis.^106^ CBD is a well‐known TRPV2 activator 8^107^, ^108^, ^109^ and is able to increase DOXO drug permeation^106^ and, as mentioned above, chemosensitivity to BCNU,^70^ in glioma cells. In summary, CBD seems to induce cell death in GSCs through various mechanisms of action, including cell cycle arrest at the G_0_/G_1_ phase,^70^ apoptosis,^67^, ^68^ and autophagy.^67^, ^70^

The synergy of CBD and DOXO has been previously evaluated, generating an increased apoptosis and a reduced metastasis. Also, CBD + DOXO can arrest cell cycle in G1 and reduce migration in MDA‐MB‐231 triple negative breast cancer cell line in an in vivo and in vitro assay.^110^ The synergic effect is also observed in glioblastoma, where CBD‐induced TRPV2 activation reduces the drug resistance of tumor cells, increasing the DOXO intake.^70^

Some of the studies in GBM evaluated the effects of combining CBD with other cannabinoids, such as CBG, THC, or the chemotherapy drug temozolomide (TMZ). Lah et al.^68^ analyzed the effect of different cannabinoids (CBG, CBD and THC), alone or combined with TMZ, on the viability and GSC lines (NCH644, NCH421k, and patient‐derived K26). CBD exhibited the highest cytotoxicity in GSCs with IC_50_ mean values of 19.5 ± 4.2 μM (15.9 μM in NCH644; 27.9 μM in NCH421k; and 14.6 μM in K26) compared to CBG and THC, with IC_50_ mean values of 59.0 ± 14.7 and 22.8 ± 3.3 μM, respectively. They described that CBD induced apoptosis in GSC line K26 with a 40–50% of apoptotic cell population. The combination of CBD:CBG (3:1 ratio) showed an additive response in GSCs (NCH644, NCH421k, K26), but the addition of THC and TMZ did not contributed to any extra cytotoxic effect on these CBD:CBG mixtures. Furthermore, in the GSC line NCH421k, they showed that the combination at 3:1 ratio between CBD and CBG significantly inhibited invasion at nearly 50%, whereas CBD alone showed no statistically significant effect.^68^ In a second publication, the same authors tested the combination of CBD and CBG (3:1 ratio) in eight GSC lines. The GSC lines treated with CBD alone had a mean IC_50_ value of 50 ± 7.1 μM, whereas the mean IC_50_ value was 42 ± 3.4:16 ± 1.1 μM when treated with the combination. In NCH644, the IC_50_ of CBD alone was 34 μM, which was the same value as in the combination of CBD:CBG (34:11 μM). The treatments of CBD and CBD:CBG were more effective against GSC than in the GBM lines, had mean IC_50_ of 78.9 ± 7.8 μM and 76 ± 6.6:25 ± 2.1 μM, respectively.^111^ On the other hand, López‐Valero et al.^69^ tested the effect of different combinations between THC, CBD and TMZ in cell viability and neurosphere formation of two glioblastoma patient‐derived glioma initiating cells (GH2‐GICs and 12O12‐GICs). Combinations of THC:CBD (1:5 ratio) and THC:CBD (1:1 or 1:5) + TMZ exhibited a higher reduction on the proliferation of GH2‐GICs than THC:CBD (1:1) or TMZ alone. Similar results were observed in 12O12‐GICs, with the strongest effect seen with THC:CBD (1:5) + TMZ. Furthermore, the combination of THC:CBD in a 1:5 ratio demonstrated greater inhibition of neurosphere formation compared to THC:CBD in a 1:1 ratio or TMZ alone. However, the most potent inhibition was observed with THC:CBD (1:5) + TMZ, which was also superior to THC:CBD (1:1) + TMZ. Similar results were obtained in vivo in intracranial orthotopic xenografts generated by injecting GICs into immunodeficient nude mice. The combination of THC:CBD (1:5) + TMZ resulted in a significant reduction in tumor growth and increased animal survival rates, surpassing the effects of THC:CBD (1:1) + TMZ or TMZ alone. However, the combination of THC and CBD (1:5) without TMZ did not yield significant results.^69^

Combination studies have shown that a higher amount of CBD in combination with CBG, THC, or TMZ produces a greater effect against GBM stem cells both in vitro and in vivo. The combination of CBD and CBG in a 3:1 ratio,^12^, ^68^ as well as THC and CBD in a 1:5 ratio with TMZ,^69^ has been found to be the most effective in inhibiting cell viability and neurosphere formation. In fact, a phase II clinical trial (ARISTOCRAT) with CBD and THC (Sativex) is currently underway for the treatment of unresectable and recurrent glioblastoma. It has been underway since 2022 and pending the publication of results.^70^

Regarding lung cancer, three articles were found in which the effect of CBD on lung tumor stem cells was evaluated. Hamad and Olsen^75^ investigated the effects of CBD treatment on sphere formation in two non‐small cell lung cancer (NSCLC) cell lines (A549 and H1299) and one small cell lung cancer (SCLC) cell line (H69). The results showed that CBD induced a significant reduction in sphere formation in A549, H69 and H1299 cells in serum‐free medium. CBD also altered CSC marker levels in treated spheres, significantly decreasing SOX2, Oct‐4 (POU5F1), and CD133 (PROM1) in A549; SOX2 and PROM1 in H1299; and SOX2, CD44, PROM1, and POU5F1 in H69. In addition, CBD increased the number of annexin‐positive cells, significantly activated caspase 3/7, upregulated the expression of TP53, CDKN1A and pro‐apoptotic genes BAK1, BAX and BAD, increased ROS levels and caused a significant reduction in mitochondrial membrane potential.^75^ The effect of CBD on sphere formation was also evaluated using two NSCLC cisplatin‐resistant (CR) cell lines (H460‐CR and A540‐CR), by Misri et al.^74^ The results showed a significant reduction in sphere formation compared to untreated or cisplatin‐treated cells. In addition, the treatment with CDB resulted in a significant decrease in the population of CD44+ and CD133+ cells in CSC spheres. Moreover, there was a significant downregulation of protein expression of Snail, Nanog, and Vimentin in both H460‐CR and A549‐CR.^74^ The reduction of CD44 expression in lung cancer cells by the action of CBD treatment was also demonstrated in vivo. Salles et al.^73^ established an in vivo lung cancer model with a subcutaneous tumor from NCI‐H1437 in nude mice to test CBD treatment. In comparison with placebo‐treated group, inhalant CBD reduced tumor growth rate and evidenced a decrease in CD44 expression by immunohistochemistry and by flow cytometry analysis with a reduction of CD44+ cells from 12.1% to 3.2% of total tumor cells.^73^ CD44 is a key cancer stem cell biomarker that promotes epithelial‐mesenchymal transition and is involved in cancer proliferation, invasion, metastasis, and resistance.^112^ In particular, it has been associated with poor prognosis and metastasis in lung cancer.^113^, ^114^ Thus, CD44 constitutes a potential therapeutic target for various cancers.^115^

Finally, only one article examined CBD's effect on breast cancer CSCs. Jo et al.^76^ found that CBD treatment inhibited MCF7‐derived mammosphere formation and growth, and significantly reduced stem cell protein expression (ALDH1A1, CD133, NANOG, SOX2) in treated mammospheres.^76^

## 4 CONCLUSIONS

This systematic review reveals the regenerative and protective action that CBD presents on the SSCs. CBD can stimulate the differentiation of SSCs into osteogenic, adipogenic and neurogenic cell lines directly by a production or inhibition of key factors involved in cellular genesis or indirectly protecting the SSCs against inflammation, oxidative stress and other harmful agents in cellular genesis, both of them without causing adverse or toxic effects. The positive effect of CBD on osteogenesis is supported by in vivo studies, demonstrating an enhanced bone regeneration with scaffold and without them, and also an improved neural stem cell response. The protective effect of CBD has been also observed in rodent schizophrenia model, disease in which SSC activity is reduced. Nowadays there is not enough clinical trials using CBD as a stem cell stimulator. Only one author studied the effect of CBD as a GVHD treatment in patients with a hematopoietic stem cell implantation, with positive results during the 30 days treatment.

Furthermore, although there are numerous articles that study the antitumor effect of CBD, there are no studies that investigate into its specific activity on CSCs. On the other hand, its effect on CSC has only been studied in three tumor types, making it necessary to extend the studies to other types of aggressive and prevalent tumors. The most studied tumor type is the GBM, in which CBD showed an increased cellular differentiation marker levels while reducing proliferation and cell viability. Moreover, CBD decreases spheroid formation and stem cell marker levels in lung and breast cancers. Among the three tumor types used as study models, only GBM and lung have been studied in vivo, demonstrating a reduction in tumor growth and an increased survival rate and a modified cellular markers expression in CBD‐treated mice.

In conclusion, CBD presents a powerful and promising activity in Regenerative Medicine as a differentiator and protector of SCCs and in Oncology as a potential antitumor treatment focused on CSCs. However, this systematic review reveals the existence of few in vivo studies. Therefore, further in vivo studies would be necessary to justify the translational leap of these pharmacological therapies to controlled, randomized clinical trials.

## AUTHOR CONTRIBUTIONS

Jose Prados: Conceptualized the manuscript. Cristina Mesas, Javier Moreno and Kevin Doello: Collected the literature, carried out the investigation and wrote the original draft. Mercedes Peña used software and supervised manuscript. Jose Prados and Juan M. López‐Romero, writing—review and editing the manuscript. Consolación Melguizo participate in funding acquisition. All authors have read and agreed to the published version of the manuscript.

## FUNDING INFORMATION

This research was funded in part by Spanish Ministry of Science, Innovation and Universities, as well as the European Union, through project RTC2019‐006870‐1. In addition, this work was supported by funds from research group CTS‐107 (Andalusian Government).

## CONFLICT OF INTEREST STATEMENT

The authors declare no conflict of interest.

## Data Availability

No data was used in the present study.
